# Different facets of age perception in people with developmental prosopagnosia and “super-recognisers”

**DOI:** 10.1186/s41235-024-00603-4

**Published:** 2024-11-13

**Authors:** Janice Attard-Johnson, Olivia Dark, Ebony Murray, Sarah Bate

**Affiliations:** 1 Department of Psychology, Faculty of Science and Technology, Bournemouth University, Fern Barrow, Poole, BH12 5BB UK; 2 Department of Psychological Sciences, School of Natural and Social Sciences, University of Gloucestershire, Cheltenham, GL50 4AZ UK

**Keywords:** Age estimation, Prosopagnosia, Super-recognisers, Face processing, Facial identity recognition, Cognitive estimation

## Abstract

The interplay between facial age and facial identity is evident from several scenarios experienced in daily life, such as when recognising a face several decades after the last exposure. However, the link between age and identity processing, and how age perception abilities might diverge in individuals with different face processing abilities, has scarcely been considered. Furthermore, the approach used to test age perception ability may also influence outcome, but the effect of different paradigms on performance is not yet known. Across three studies, we compare super-recognisers (SRs), people with developmental prosopagnosia (DPs), and a group of neurotypical controls, on three age perception paradigms. There were no differences on the numeric age estimation task (i.e. providing precise age estimates for a series of faces; Study 1), and numeric age estimation task with added noise-distortion to stimuli (Study 2). However, SRs were more accurate when instructed to classify ambient faces as either over- or under- the age of 18 compared to both DPs and controls (Study 3). Thus, there may be nuanced differences in age processing which can be tapped into using separate paradigms; however, given that the difference is only with SRs it remains unclear to what extent these are linked to facial identity processing.

**Supplementary Information:**

The online version contains supplementary material available at 10.1186/s41235-024-00603-4.

## Introduction

The visual processing of faces has been intensely researched over the past 3 decades, with a considerable body of work concentrating on the perception and recognition of facial identity (see Bate, 2013; Bindemann, 2021; Bruce & Young, 1986, 2012; Hole & Bourne, 2010; Johnston & Edmonds, 2009). Although identity recognition is a core aspect of face processing, the face also portrays a plethora of non-identity information, such as facial expression, sex, personality, and age. Relationships between the former three and facial identity have been explored to varying degrees (e.g. Bate & Bennetts, 2015; Marsh et al., 2019; Rossion, 2002; Redfern & Benton, 2017; Satchell et al., 2019), however, far less work has examined the potential link between age and identity perception. This is surprising considering the ostensible interplay between facial age and facial identity in daily life. For example, the natural ageing process can produce fundamental changes to the face which may influence one’s ability to identify a face when encountered again several years later (George & Hole, 1998; Mileva et al., 2020; Sexton et al., 2024). Thus, it follows that the ability to accurately perceive and evaluate age-related facial features may be associated with face identification ability.

Existing theoretical and neurological models of face processing largely omit age perception, and thus also offer few clues as to how facial identity and age perception relate to one another. One of the earliest cognitive models of face processing (Bruce & Young, 1986) suggests that non-identity specific information which can be derived from physical features of a perceived face (such as ethnicity, gender, expression, and personality attributes) can be useful in remembering unfamiliar faces but generally operates independently of identity recognition. These were afforded the term *visually derived semantic codes,* and age was included in this category. Subsequent cognitive (Burton et al., 1999; Valentine, 1991) and neurological (Duchaine & Yovel, 2015; Gobbini & Haxby, 2007; Haxby et al., 2000) models omit age perception entirely. Therefore, these theoretical frameworks offer limited hypotheses about the processing of facial age, unless assumptions are extended from other non-identity facial characteristics which have received relatively more attention (such as emotional expression and sex perception) (e.g. Calder & Young, 2016; Chen et al., 2022).

However, conflating findings from other aspects of non-identity perception may be inappropriate because age is a unique aspect of the face that is subjected to slow and progressive changes to facial features over the course of the lifespan. This is distinctly different from emotional expression, which transiently conveys social communication and changes during a single interaction; and sex, which remains relatively stable over the lifespan. Further, emotional expression processing draws upon a complex neural network (Adolphs, 2002; Haxby & Gobbini, 2011), and it is difficult to disentangle perceptual from affective processes. In contrast, the perception of age offers a more favourable opportunity to isolate identity from non-identity perception without confounds from other processes, and therefore an innovative opportunity to progress the theoretical debate surrounding the specificity of facial identity recognition within the wider face processing system.

A key feature of research examining facial identity recognition is the profound individual differences that have been observed in the skill (e.g. Bindemann et al., 2012; Bruce et al., 2018; Stantic et al., 2022). That is, a wide distribution in the ability to identify faces ranges from individuals with clinical difficulties in recognising faces (developmental prosopagnosia, DP; Bate & Tree, 2017; Stantic et al., 2022) to others who excel at the task (super-recognisers, SRs; Bate et al., 2018; Bobak et al., 2016). Yet, very little work has examined whether a similar spectrum of ability also exists for the perception of age, and, if so, how it maps onto that for face recognition ability. Findings from a single case study (Nunn et al., 2001) and small groups of individuals with developmental prosopagnosia (DP) (Chatterjee & Nakayama, 2012; Dobel et al., 2007) suggest that impairments in the domain of facial identity do not translate to deficits in age perception. However, impaired performance on an age task has been recorded in a small number of single case studies of acquired prosopagnosia (Thomas et al., 2008; Tranel et al., 1988). More fundamentally, the assessment tools available for the study of age perception are severely lacking in comparison with those that are used to assess facial identity processing, where researchers have free access to a number of appropriately-calibrated psychometric-standard assessment tasks (e.g. the Cambridge Face Memory test, CFMT, Duchaine & Nakayama, 2006; Cambridge Face Memory test—long form, CFMT+, Russell et al., 2009; Cambridge Face Perception Test, CFPT, Duchaine et al., 2007). Indeed, existing tasks that have been used to investigate age perception may not be adequately sensitive to capture any nuances in difficulties with respect to different aspects of the process. This can be illustrated by the ceiling performance of 100% accuracy in an age sorting task recorded in all control and DP participants (Dobel et al., 2007), and 90% performance accuracy for an age discrimination task (Thomas et al., 2008), both of which indicate that a more sensitive test is needed. Further, the small number of existing studies have also differed in their use of natural faces (Dobel et al., 2007; Nunn et al., 2001) versus those that have been artificially generated (Chatterjee & Nakayama, 2012; Thomas et al., 2008). In the facial identity literature, there is some evidence to suggest that artificially generated faces do not accurately reflect face processing abilities compared to real faces (e.g. Balas & Pacella, 2015; Crookes et al., 2015), and it is possible the same limitation may extend to age perception.

There may also be differences in ability according to the type of task being completed. For example, estimating the age of a face by providing a precise numerical age may be a qualitatively different task to those requiring the comparison of pairs of faces to determine relative age (e.g. discriminating older or younger faces) (Nunn et al., 2001; Thomas et al., 2008), the ordering of faces by perceived age (Chatterjee & Nakayama, 2012; Dobel et al., 2007), or the simple classification of faces as child or adult. Numerical estimation of age, for example, may draw on the ability to compare the target face with an existing internal representation of faces in different age categories, which may also be moderated by the observer’s degree of exposure to faces of known ages across the lifespan. The process of comparing a new ‘problem’ with existing knowledge or an internal model to provide a ‘best guess’, is a prominent aspect of cognitive estimation used in other more widely researched tasks such as estimation of weight, height, time, and distance (Shallice & Evans, 1978; Wagner et al., 2011). Cognitive estimation has been strongly linked with a set of higher-order cognitive functions within Executive Functioning (MacPherson et al., 2014; Shallice & Evans, 1978; Wagner et al., 2011), and therefore it is plausible that numerical estimation of age also draws on EF processes. Contrast this with the task of ordering faces by age in Chatterjee and Nakayama’s (2012) work whereby participants ordered ten sets of 6 artificially aged faces using a narrow age range (30–45 years) depicting subtle differences in age. This process may rely more heavily on perceptual abilities, given that the task involves detecting minor changes in the face and comparing these differences with available visual information. Other approaches to measuring age may draw on a combination of perceptual and EF abilities. For example, determining whether a face is that of a child or adult (e.g. under/over age of 18) may also require an internal representation of these age groups, but perhaps also a perceptual ability to detect subtle differences in facial features where more apparent changes associated with aging later in life (e.g. wrinkles, skin tone) are not available. Given the potential differences in processing which may underlie each of these tasks, proficiency in one task may not extend to all measures.

Finally, in addition to the theoretical merit of examining age perception, there is also considerable applied value. The task of estimating age, specifically when identifying documentation is unavailable, is critical in areas relating to immigration and law enforcement (e.g. for the assessment of asylum seekers requiring classification of children or adults, or the identification of victims in sexually explicit photographs). The consequences of misclassifying a child as an adult (or vice versa) in these security settings can be serious. If some individuals are particularly proficient at such tasks, they could be mobilised in a similar manner to the recent use of SRs in policing settings for the purposes of facial identification (Dunn et al., 2023). Whether the same SRs have skills that extend to the processing of facial age is currently unknown. Indeed, some studies have found that not all SRs excel at both the memory and perception of facial identity (Bate et al., 2018, 2019), suggesting a more nuanced approach in screening is required. While a logical hypothesis is that age perception skills would have a stronger association with facial identity perception skills than face memory performance, it is also possible that the generalised process of face perception may further dissociate between identity and age. Establishing whether a broad spectrum of age perception ability exists, and whether this maps onto known differences in face identification ability, is therefore valuable for decision making around the use of SRs for age-related tasks or, if a distinct group is identified, for forming a novel classification of ‘super-age-estimators’.

The aim of this study is twofold. First, we aim to compare individuals with known differences in their face recognition ability (controls, DPs, and SRs) on their ability to estimate age from faces. Second, we examine whether there are different aspects to the perception of age, and whether these overlap with face recognition skills. To this end, an initial study assessed age perception ability in DPs, SRs and controls, using a straightforward numeric age estimation paradigm composed of high-quality images of faces from across the life span. A second study adopted the same paradigm but increased the difficulty of the task by applying noise-distortion to a new set of images. Noise-distortion specifically disrupts the visibility of skin tone and texture which are important for estimation of age (González-Alvarez & Sos-Pena, 2023; Porcheron et al., 2013), and therefore allowed us to investigate each participant group’s ability to use structural information for facial age judgments. Pertinently, unique skin characteristics can also act as markers for facial identification, for example, observers spontaneously use moles to perform a facial matching task (Fysh & Bindemann, 2022). Furthermore, qualitative evidence suggests a disproportionate reliance on such distinctive features in DPs as a compensatory mechanism for facial identity recognition (Adams et al., 2020; Murray et al., 2018; Portch et al., 2023). Thus, DPs may use similar sources of information for age estimation as opposed to structural cues. We opted for the noise-distorted approach (as opposed to pixelation) as a way to increase difficulty to maintain consistency with the CFMT+, a validated measure of facial identification (Russell et al., 2009). However, these lower-resolution images also shift slightly the ecological validity of the task towards some real-world security settings by increasing difficulty. Specifically, lower-resolution images (grainy or pixelated images), or partially obscured through other graphic manipulations or poor camera resolution, are likely to be encountered when assessing online pornographic materials. A final study then maximised the ecological validity of the investigation, by directly simulating the most commonplace scenario in professional settings (i.e. sales of restricted items, classification of asylum seekers as minors or adults, and assessing online explicit materials for minors), which is to classify faces of individuals aged between 14 and 22 as being over- or under- the age of 18.

## Study 1

In study 1, the ability of controls, SRs and DPs to provide a numerical estimate of age from faces was assessed. Numerical estimation of age may draw on the ability to compare the target face with an existing internal representation of faces in different age categories. Some evidence suggests that the internal facial representations in at least some DPs are intact (Bate et al., 2008), but the connection with perceptual encoding and semantic systems is severed (Fox et al., 2008). Consequently, DPs may be unable to access facial representations for identity recognition, and if this is the case, it might be predicted that they are also unable to use stored representations for other aspects of processing. The flip of this is that, if SRs are more proficient at facial identity recognition because they are better able to recall stored facial representations, they might also be proficient at the current task. For this study, photographic images of male and female faces from across the adult life span with known ages (20 to 60 years) were selected from an existing database (Minear & Park, 2004).

## Method

### Participants

A total of 116 participants were recruited, however three participants were removed because they did not provide the appropriate information or completed the task twice. Of the remaining 113 participants who completed all tests, 31 (26 female) individuals had previously passed a pre-screening for DP, (*M* = 46 years, range 22–61, SD = 12.03), and 33 (23 female) were known SRs (*M* = 42 years, range 23–63, SD = 9.93) (full screening data for DPs and SRs are provided in supplementary material). In addition, 49 (23 female, 1 other) matched control participants (33 female) were recruited (*M* = 40 years, range 19–74, SD = 12.63). A one-way ANOVA confirmed that participant age did not differ significantly across the three groups, *F* (2, 110) = 2.81, *p* = 0.064. All but seven participants selected ‘White’ for their ethnicity, while two selected ‘South Asian’ (SRs), one selected ‘Hispanic’ (SR) and four selected ‘Other’ (2 DPs and 2 SRs). All participants were compensated for their time. Ethical approval was obtained from the Institution’s Ethics Board (Ref: 47626), and all participants provided written consent to take part.

### Materials

**Fig. 1:**
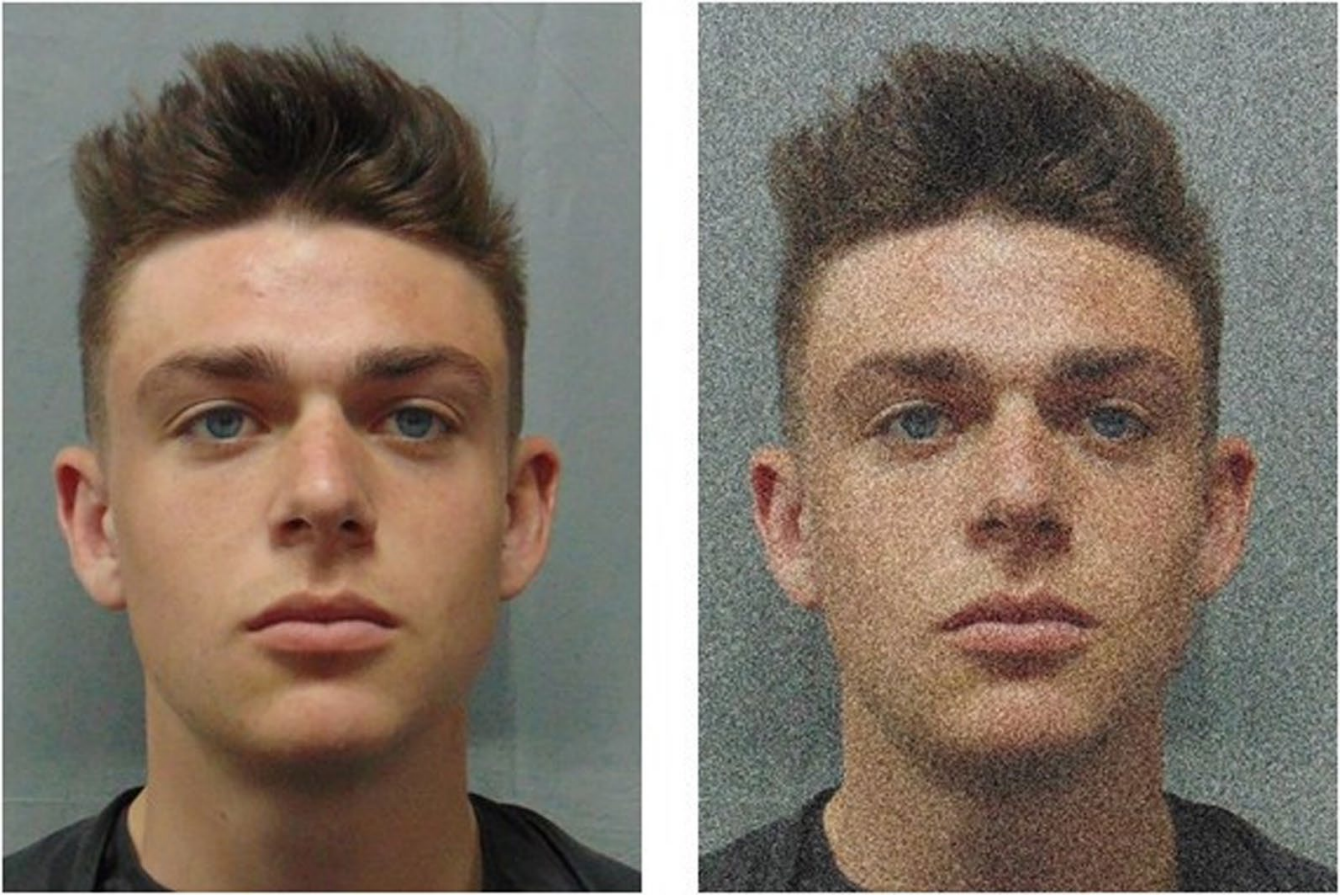
Example image of original and noise-distorted images from Studies 1 (left) and 2 (right). Due to restrictions in permissions to reprint photographs from the database used in the actual studies, these images are a representative example using similar images (taken from Murray et al., 2022 database) for illustrative purposes and not the ones used in these studies

A total of 30 images were selected from the Minear and Park (2004) face database and these comprised 15 male and 15 female images which were spread equally between the five following age categories: 20s, 30s, 40s, 50s, 60s. All faces were Caucasian, frontal profile, had no external accessories (e.g. glasses, jewellery), and had neutral facial expressions (for an example, see Fig. 1). The images were 480 pixels in height, and the width varied slightly around 640 pixels to ensure that the aspect ratio of the image was not distorted.

### Procedure

The experiment was conducted online using an established online testing platform *Testable* (testable.org) which uses a robust calibration feature to adjust the experiment to the size of the participant’s monitor. The task could only be accessed on a computer, and it was not possible to take part using a tablet or mobile device. *Testable* has been frequently used in published work with controls, DPs, and SRs (e.g. Murray et al., 2022). Participants were instructed to estimate the age of each face displayed as precisely as possible and then enter the age using the keyboard. Each face was presented in the centre of a blank white screen until a response was made but up to a maximum duration of 5000 ms after which the face was removed from view. Faces were presented in a random order for each participant.

### Transparency and openness

We report how we obtained our sample, all data exclusions, all measures and manipulations in the study. The datasets generated and analysed during the current study are available in the Open Science Framework repository (URL: https://osf.io/t3dv2/?view_only=17d695679fec4d4cb61eb5d47059f98f). Data were processed using RStudio (version 2023.03.0) and the package *tidyverse*, and data analysed using JASP. The study’s design and its analysis were not pre-registered. The year of data collection was 2023.

## Results

### Data processing

For each participant, responses on trials which had timed out (i.e. those with response times over 5000 ms) were removed. As is typical in age estimation research using similar paradigms (e.g. Cliffard et al., 2018; Davis & Attard-Johnson, 2022; Voelkle et al., 2012), for each of the remaining trials, a difference score was calculated by subtracting *actual age* from *estimated age* (estimated age—actual age) for each participant. To calculate a score for *Estimation Bias,* this difference score was then averaged across each of the five stimulus age categories (i.e. 20–29, 30–39, 40–49, 50–59, 60–69). A positive score indicates a bias towards overestimating the age of the faces, and a negative score reflected an under-estimation. However, Estimation Bias may not detect inaccuracies in estimation which are not systematically directional as large over- and under- estimates may negate each other when averaged to provide a false ‘accuracy’ (Voelkle et al., 2012). To address this, we also calculated *Absolute Accuracy,* which reflects the overall extent of estimation accuracy without direction (see Clifford et al., 2018; Davis & Attard-Johnson, 2022; Voelkle et al., 2012).

Accuracy is the primary measure of interest in all the tasks reported in the series of studies presented here, and for this reason participants were asked to respond as accurately as possible, and not as quickly as possible. However, to provide a complete picture and detect any nuances in differences between groups, as well as to detect any potential speed-accuracy trade-offs (Fysh & Ramon, 2022), response time data will be reported and analysed to complement the accuracy data.

Mixed factor ANCOVA and the analogous Bayesian analysis were conducted and are reported to quantify evidence for the alternative and null hypotheses. All analysis was performed in JASP (version 17.2) using the default prior. We report Bayes Factor 10 (BF_10_) for main effects which represents how likely it is for the data to arise under the alternative model compared to the null model. For the interaction term, we report the effects for the Inclusion Bayes Factor (BF_inc_) across matched-models which reflects the evidence for the interaction model stripped of other effects (see Mathôt, 2017). A Bayes Factors of greater than 3 will represent substantial support for the alternative hypothesis and smaller than 0.3 will represent substantial support for the null hypothesis, and a BF in-between 0.3 and 3 represents only weak or anecdotal evidence (Jeffreys, 1961; Wetzels et al., 2011).

### Reliability analysis

To assess the internal reliability of the Numeric Estimation Task a split-half reliability analysis was performed on the *Estimation Bias* score. For this, trials were split into two sets separated by odd and even trials and compared. 56 cases were excluded due to missing or erroneous data, and the remaining 59 included in the analysis. The two sets were found to be positively correlated, *r* = 0.661, *p* < 0.001, with a Cronbach’s alpha of 0.734, and a Spearman-Brown Coefficient of 0.796 which are considered good.

### Estimation bias

**Fig. 2:**
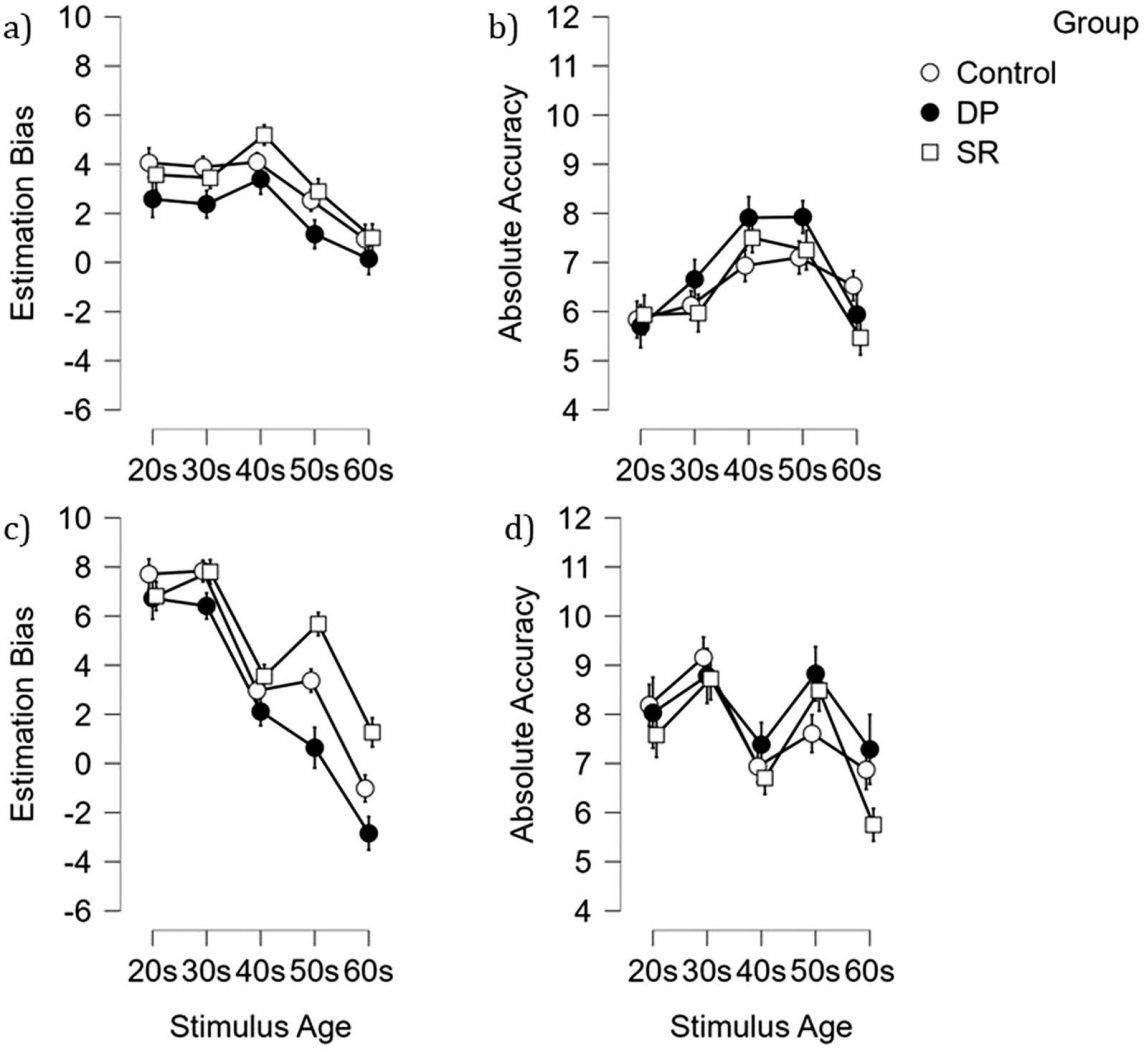
Illustration of mean deviation from true target age (in years) separate for all five stimulus age categories for high-resolution images (Study 1) representing Estimation Bias and Absolute Accuracy (**a** and **b**, respectively), and for noise-distorted images (Study 2) also representing Estimation Bias and Absolute Accuracy (**c** and **d**, respectively). Bars represent Standard Error of the mean

Figure 2a illustrates the average Estimation Bias separated by participant group for each stimulus age group. A 3 (Group: controls, SRs, DPs) × 5 (Stimulus age: 20s, 30s, 40s, 50s, 60s) mixed factorial ANCOVA on Estimation Bias, controlling for participant age entered as a covariate, was performed.^1^ The covariate for age was found to be significant, *F*(1,109) = 9.30, *p* = 0.003, *ƞp*^2^ = 0.08, BF_10_ = 25.28.

Therefore, when participant age is controlled for, this analysis found no main effect of group, *F*(2,109) = 0.62, *p* = 0.543, *ƞp*^2^ = 0.011, BF_10_ = 0.24. No interaction between group and stimulus age was found, *F*(8,436) = 0.36, *p* = 0.941, *ƞp*^2^ = 0.007, BF_inc_ = 0.004 demonstrating extreme evidence in favour of the null model over the model with Group x Stimulus age. However, a main effect of stimulus age was revealed, *F*(4,436) = 4.14, *p* = 0.003, *ƞp*^2^ = 0.037, BF_10_ = 1.51 × 10^11^.

Bonferroni adjusted post hoc comparisons compared against an alpha threshold of 0.005 (*p* value adjusted for comparing a family of 10, 0.05/10) revealed that across all participant groups, Estimation Bias was lowest (closest to zero) for stimuli depicting faces aged in their 60 s compared to all other age groups, all *t*s ≥ 4.51, all *p*s ≤ 0.001, and all *d*s ≥ 0.42. Faces in their 50 s also yielded lower Estimation Bias compared to faces in their 40 s, *t*(112) = 5.18, *p* < 0.001, *d* = 0.49. No other comparisons reached significance, all *t*s ≤ 2.52, all *p*s ≥ 0.013, all *d*s ≤ 0.23.

Together, these data suggest that face recognition ability is not related to any systematic pattern (i.e. systematic and consistent over- or under-estimation of age) of responding when providing a numeric estimate of the facial age of faces across the adult lifespan (20–60-year-olds). However, it is possible that differences emerge when considering overall accuracy.

### Absolute accuracy

Figure 2b illustrates the average Absolute Accuracy separated by participant group for each stimulus age group. Again, a mixed ANCOVA was performed, however this time with Absolute Error as the dependent variable. The covariate of participant age was not found to be significant, *F*(1,109) = 0.05, *p* = 0.824, *ƞp*^2^ = 0.00046, BF_10_ = 0.15.

This analysis revealed a main effect of stimulus age, *F*(4,436) = 2.42, *p* = 0.048, *ƞp*^2^ = 0.022, BF_10_ = 3.56 × 10^6^. However, sphericity was violated, and the main effect was no longer significant when Greenhouse–Geisser correction was applied [*F*(3.69,405.84) = 2.42, *p* = 0.053]. The main effect of participant group, *F*(2,109) = 0.57, *p* = 0.568, *ƞp*^2^ = 0.01, BF_10_ = 0.082, and interaction between stimulus age and group, *F* (8,436) = 1.16, *p* = 0.32, *ƞp*^2^ = 0.02, BF_inc_ = 0.037, were also not significant.^1^

Together, these data suggest that the ability to provide an accurate numeric estimation of the age of adult faces is likely to be independent of face recognition abilities.

## Discussion

Study 1 examined whether controls, DPs, and SRs differ in their ability to estimate the age of faces across the lifespan and the task devised for this purpose was found to have a good range of reliability (De Vet et al., 2017). Findings indicated that there were no group differences in the ability to provide numerical estimates of the faces, and Bayesian Analysis found moderate evidence in support of the null hypothesis. There were some differences in Estimation Bias across the different stimulus ages, with a pattern suggesting that the ages of older faces (60+) were less vulnerable to systematic bias in age perception achieving an overall bias of close to zero (0.7 years), relative to the younger faces (20-, 30-, and 40-year-olds) which had a tendency to be perceived on average 4–5 years older.

With regard to overall accuracy (i.e. Absolute Accuracy), DPs once again performed similarly to controls and SRs, with Bayesian Analysis demonstrating strong evidence for the null effect. These findings suggest that DPs may be able to access stored representations of faces, at least for the purposes of age perception. It is also possible that age perception relies less on structural properties of the face but more on cosmetic features of the face, such as skin tone, texture, blemishes etc. Research suggests that DPs may rely more heavily on these cosmetic aspects (Adams et al., 2020; Murray et al., 2018; Portch et al., 2023) and may therefore be attuned to detecting such subtle differences which may serve an additional advantage for the purpose of age perception. Thus, for the next study, we examined this possibility by reducing the availability of the visual information relating to skin texture and tone.

## Study 2

Study 2 examined whether DPs may be more susceptible to a reduction in the image quality of the photographs. High-resolution images reveal visual information related to fine lines and skin texture which are important for age cues, and it is unclear how disrupting this information affects age estimation accuracy in controls, DPs, and SRs. Distinctive skin features can act as markers for facial identification, for example, observers spontaneously use moles to perform a facial matching task (Fysh & Bindemann, 2022). Indeed, much anecdotal evidence suggests that disproportionately DPs rely on distinctive features such as blemishes to identify faces (Adams et al., 2020; Murray et al., 2018; Portch et al., 2023), and they may use similar sources of information for age estimation as opposed to structural cues. To this end, a paradigm identical to Study 1 was used with a different set of images from the same database. However, to more closely reflect the quality of the images which may also be encountered in the real-world, these images were modified by adding noise using a similar approach to the ‘noise-distorted’ condition in the CFMT+ (Russell et al., 2009).

## Method

The same participants took part as in Study 1 and completed the task after Study 1. Visual noise was added to an additional set of 30 images selected from the Minear and Park (2004) database, also comprising 15 male and 15 female images which were spread equally between the five following age categories: 20s, 30s, 40s, 50s, 60s. As before, all faces were Caucasian, frontal profile, had no external accessories (e.g. glasses, jewellery), and had neutral facial expressions. The images were 480 pixels in height, and the width varied slightly around 640 pixels. Noise was added to the images by applying 30% Gaussian noise (monochromatic) in Photoshop CC (version 20) (see Fig. 1). using a similar approach to the noise-distorted images in the CFMT+ (Russell et al., 2009) described in previous work (Arrington et al., 2022).

### Procedure

The experiment was conducted online using Testable. Participants were instructed to estimate the age of the face as precisely as possible and then enter the age using the keyboard. Each face was displayed in the centre of a blank white screen, with the images measuring 480 pixels in height, until a response was made up to a maximum duration of 5000 ms. If a response was not made, the image was removed from display and moved on to the next trial.

## Results

### Reliability analysis

To assess the internal reliability of the Numeric Estimation Task a split-half reliability analysis was performed on the *Estimation Bias* score. For this, trials were split into two sets separated by odd and even trials and compared. 31 cases were excluded due to missing or erroneous data, and the remaining 85 included in the analysis. The two sets were found to be positively correlated, *r* = 0.826, *p* < 0.001, with a Cronbach’s alpha of 0.905, and a Spearman-Brown Coefficient of 0.905.

### High-resolution versus noise-distorted images

To determine whether the noise-distorted image task was more difficult than the one used in Study 1, we compared overall Absolute Accuracy for the high resolution and noise-distorted images collapsed across all stimulus age groups. A 3 (Group: control, SRs, DPs) × 2 (Image quality:

high resolution, noise-distorted) mixed factorial ANOVA revealed a main effect of image quality, *F*(1,110) = 55.23, *p* < 0.001, *ƞp*^2^ = 0.334, BF_10_ = 1.12 × 10^9^, whereby accuracy was poorest for the noise-distorted image set with a mean deviation from actual age of 7.74 years compared to 6.57 years for the high-resolution images. No main effect of group or interaction was found, *F*(2,110) = 0.85, *p* = 0.430, *ƞp*^2^ = 0.015, BF_10_ = 0.21, and *F*(2,110) = 0.27, *p* = 0.767, *ƞp*^2^ = 0.005, BF_inc_ = 0.20, respectively.

### Estimation bias

The mean Estimation Bias for the different participant groups for the noise-distorted images is summarised in Fig. 2c. A 3 (Group: controls, SRs, DPs) × 5 (Stimulus age: 20 s, 30 s, 40 s, 50 s, 60 s) mixed factorial ANCOVA, controlling for participant age, was performed.^1^ The covariate of participant age was found to be significant, *F*(1,109) = 4.22, *p* = 0.043, *ƞp*^2^ = 0.037, BF_10_ = 2.11. A main effect of group was not found, *F*(2,109) = 2.08, *p* = 0.130, *ƞp*^2^ = 0.037, BF_10_ = 0.63. However, the analysis revealed a main effect of stimulus age, *F*(4, 436) = 11.84, *p* < 0.001, *ƞp*^2^ = 0.098, BF_10_ = 6.40 × 10^58^, and an interaction between stimulus age and participant group, *F*(8, 436) = 3.03, *p* = 0.003, *ƞp*^2^ = 0.054, BF_inc_ = 4.52.

To analyse the interaction effect, each participant group (control, SR, DP) were compared to each other for each age category resulting in a total of 15 independent samples *t*-tests. The alpha threshold was manually adjusted to 0.003 using Bonferroni correction method (0.05/15) and all *p* values were compared against this new alpha level. This analysis only found one significant comparison, whereby the SR group recorded greater over-estimation of age for faces in their 50 s (*M* = 5.6 years) compared to DPs (*M* = 0.6 years), *t*(62) = 3.32, *p* = 0.002, *d* = 0.83. All other comparisons were not significant, all *t*s ≤ 2.82, all *p*s ≥ 0.006, and all *d*s ≤ 0.71.

### Absolute accuracy

Data are illustrated in Fig. 2d. The mixed factorial ANCOVA with participant age as a covariate was performed again.^1^ The covariate of age was not significant, *F*(1,109) = 0.36, *p* = 0.55, *ƞp*^2^ = 0.003, BF_10_ = 0.15. The main effect of group, *F*(2,109) = 0.96, *p* = 0.385, *ƞp*^2^ = 0.017, BF_10_ = 0.10, and the interaction, *F*(8,436) = 0.78, *p* = 0.618, *ƞp*^2^ = 0.014, BF_inc_ = 0.02, were not significant. However, there was a main effect of stimulus age, *F*(4, 436) = 4.46, *p* = 0.002, *ƞp*^2^ = 0.039, BF_10_ = 4.10 × 10^6^.

Post hoc paired *t*-tests were performed with the alpha threshold adjusted to 0.005 (Bonferroni adjustment for 10 comparisons, 10/0.05). This revealed that faces in their 50 s, 30 s, and 20 s yielded more inaccurate estimates compared to faces in their 60 s (*t*(112) = 4.64, *p* < 0.001, *d* = 0.44, *t*(112) = 5.20, *p* < 0.001, *d* = 0.50, and *t*(112) = 3.13, *p* = 0.002, *d* = 0.29, respectively). Those in their 50 s and 30 s also produced more inaccurate estimates compared to faces in their 40 s (*t*(112) = 4.08, *p* < 0.001, *d* = 0.38, and *t*(112) = 6.14, *p* < 0.001, *d* = 0.58, respectively). No other comparison was found to be significant, all *t*s ≤ 2.70, all *p*s ≥ 0.008, all *d*s ≤ 0.25.

## Discussion

Study 2 examined whether the addition of visual noise to the faces affects age estimation accuracy in controls, DPs, and SRs. A single interaction was recorded for Estimation Bias which revealed that SRs overestimated the ages of those in their 50 s compared to DPs, however no other differences emerged between the participant groups. This indicates that differences in systematic patterns of age estimation are minimal. The measure Absolute Accuracy also did not reveal any group differences. However, results did show general differences in accuracy and bias across the different stimulus age groups. Generally, the faces depicting the younger age groups (20 s and 30 s) were systematically perceived as older.

These findings suggest that there are limited differences in numeric age estimation across controls, DPs, and SRs and the Bayesian Analysis provided moderate to strong evidence in support of the null hypothesis for Absolute Accuracy but was only anecdotal for Estimation Bias. However, there are multiple approaches to assessing age perception which may tap into different processing modalities. In this task, age estimation ability was measured by asking participants to provide a numeric estimate of age. Numerical estimation may draw heavily on additional non-face skills which are required for other known cognitive estimations, such as the estimation of distance, size, weight etc. These cognitive estimations have been found to rely heavily on the use of executive functioning (EF) ability (MacPherson et al., 2014; Shallice & Evans, 1978; Wagner et al., 2011). Therefore, it is possible that the task of numeric age estimation also relies more on Executive Functioning abilities and less on face processing abilities. Thus, in the next study we omitted the requirement to provide a numerical estimate. In addition, we used a task which is more akin to a real-world scenario involving the ability to discriminate adult from child. Therefore, participants were asked to classify a series of ambient faces, with no manipulation to the stimuli, as being either under- or over- the age of 18.

## Study 3

Study 3 investigated whether controls, DPs and SRs perform differently on a task which requires classification of faces as under- or over- the age of 18. This task is most analogous to a real-world scenario in professional and security settings, yet no known existing studies that have used this approach to assess age perception ability. To directly measure the relationship between relationship of this task and performance on a face identity perception task, we also included a facial identity matching task (Pairs Matching Test, Bate et al., 2018).

## Method

### Participants

The same 113 participants as in Study 1 and 2 took part in this study, the task was completed last within the same testing session. Ethical approval was obtained from the Institution’s Ethics Board (Ref: 47626), and all participants provided written consent to take part.

### Materials

#### Age classification task (ACT)

**Fig. 3:**
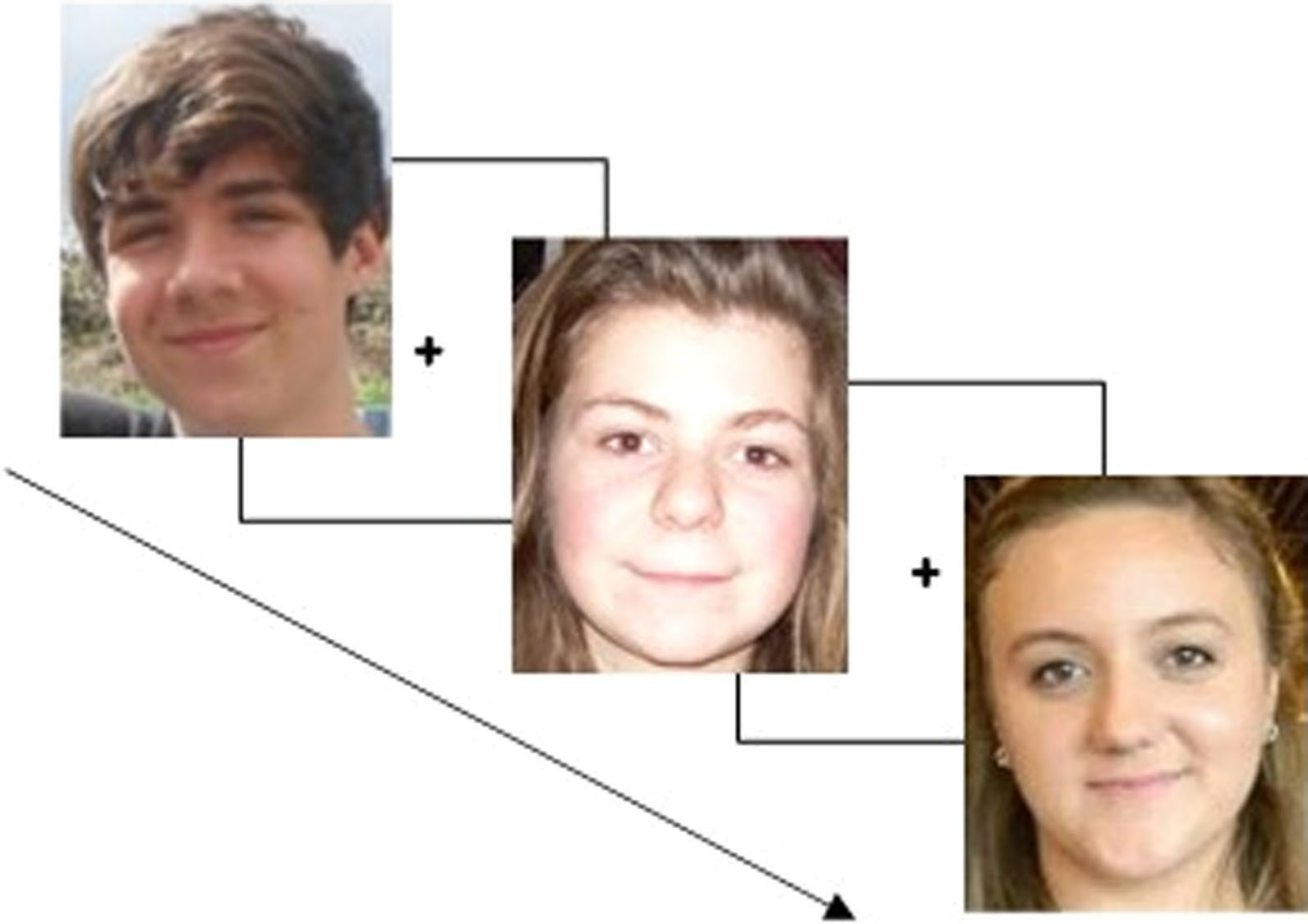
Example of the ambient face images used (from Murray et al., 2022), and an illustration of the dichotomous judgement task where participants must classify the face as being ‘over’ or ‘under’ the age of 18

A total sub-set of 32 faces were selected from our existing database (Murray et al., 2022). This set comprised 16 male and 16 female faces of identities aged between 14 and 21 (excluding the age of 18). Half of the male and female faces were under the age of 18. Faces were all in colour and varied naturally but they were all front profiles and cropped around the head to exclude the neck (see Fig. 3). Images were resized to a set width of 230 pixels, though the height was allowed to vary to ensure that the images did not become distorted. In the task, the images are displayed in the centre of a white screen. Participants are instructed to classify the faces as accurately as possible by pressing the ‘u’ key on the keyboard if they perceived the face to be under the age of 18, or ‘o’ to classify the face as over the age of 18. Images were presented only once until a response was made, and the order was automatically randomised for each participant.

#### Pairs matching test (PMT)

A pre-existing face matching test was included in this study. The PMT has an appropriate reliability of 0.74–0.79 (Bate et al., 2021). This test uses 48 colour pairs of faces (24 male), half of which are matched in identity (Bate et al., 2018). The faces are natural and include all external features. The faces in the mismatched conditions are paired according to perceived resemblance. For this task, participants view both faces simultaneously and decide whether the faces represent the same individual or two different individuals. This task is self-paced, and trials were randomly presented for each participant.

### Procedure

The experiment was conducted online using Testable. Participants completed the Age Classification Task followed by the Pairs Matching Test (PMT). The order was kept consistent across participants.

## Results

### Age classification task

#### Reliability analysis

To assess the internal reliability of the Age Classification Task, a Kuder Richardson (KR -20; Kuder & Richardson, 1937) was performed using a KR-20 calculator (Cogn-IQ.org, 2023). KR-20 is used to assess the internal consistency of measures with items with only two possible responses (i.e. correct/incorrect). The KR-20 score was found to be 0.39 which is considered low in reliability (Brennan, 2006).

#### Accuracy

**Fig. 4:**
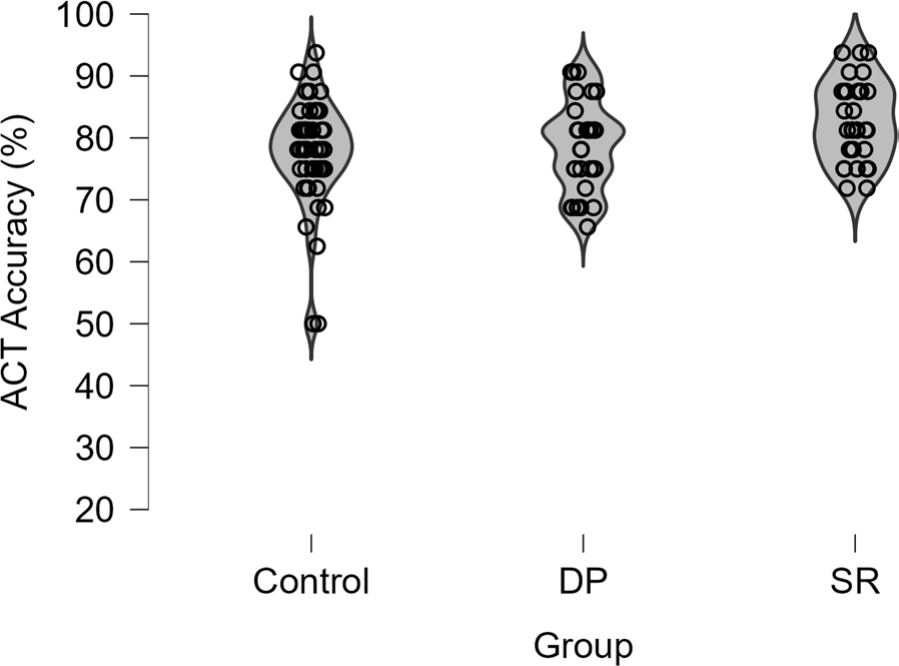
Violin plot depicting the distribution of individual percentage scores for the Age Classification Task across the control group, DPs, and SRs

Correct responses were summed, and percentage accuracy calculated for each participant. Figure 4 illustrates the distribution of percentage accuracy across the different participant groups. A one-way ANCOVA comparing participant groups (control, SRs, and DPs), controlling for participant age by entering it as a covariate, was performed. The covariate of participant age was significant, *F*(1,109) = 7.70, *p* = 0.007, *ƞp*^2^ = 0.066, BF_10_ = 3.54. This analysis revealed a significant effect of participant group, *F*(2,109) = 5.16, *p* = 0.007, *ƞp*^2^ = 0.087, BF_10_ = 3.34. Follow-up independent *t*-tests showed that SRs (*M* = 82.7%, SD = 6.4) were 4.1% more accurate compared to DPs (*M* = 78.6%, SD = 7.18), *t*(62) = 2.44, *p* = 0.018, *d* = 0.61, and 5% more accurate compared to controls (77.7%, SD = 8.6), *t*(80) = 2.87, *p* = 0.005, *d* = 0.65. DPs and Controls did not differ, *t*(78) = 0.48, *p* = 0.633, *d* = 0.11. This demonstrates a small advantage for SRs on this task, but DPs did not fare any worse than the control group.

#### Response times

Only trials with response times between 200 and 5000 ms were included in the calculation of the participant average. As above, an ANCOVA comparing participant groups (control, SRs, and DPs), controlling for participant age by entering it as a covariate, was performed. Again, there was a significant effect of the covariate, *F* (1,109) = 18.26, *p* < 0.001, *ƞp*^2^ = 0.143, BF_10_ = 2735. This analysis revealed an effect of participant group, *F* (2,109) = 17.82, *p* < 0.001, *ƞp*^2^ = 0.246, BF_10_ = 441,962, such that the control group responded faster (*M* = 1372 ms) compared to DPs (*M* = 1918 ms), *t*(78) = 5.63, *p* < 0.001, *d* = 1.29, and SRs (M = 1854 ms), *t*(80) = 4.99, *p* < 0.001, *d* = 1.12. DPs and SRs did not differ, *t*(62) = 0.59, *p* = 0.55, *d* = 0.15.

### Pairs matching task

#### Accuracy

**Fig. 5:**
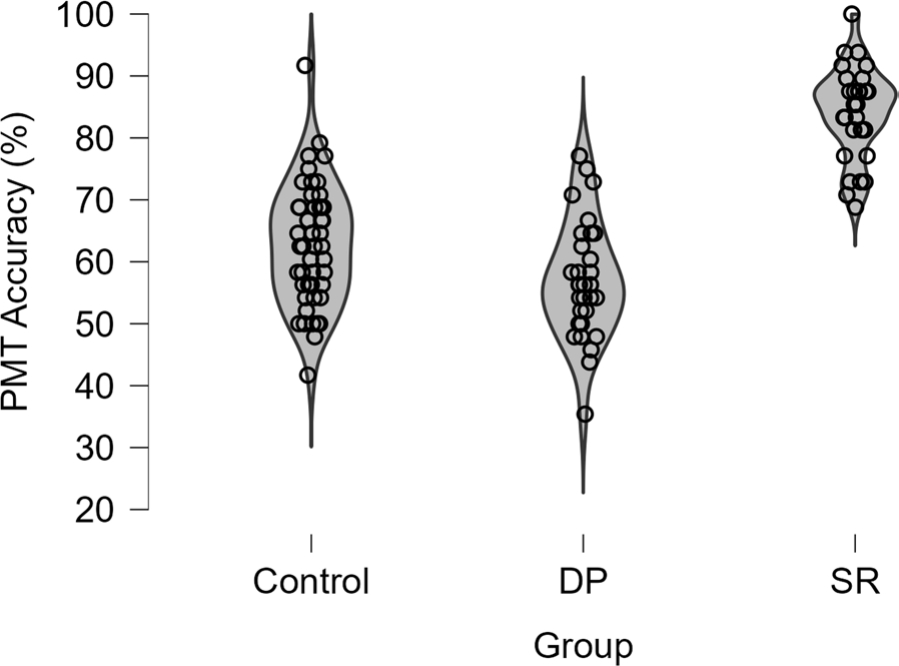
Violin plot depicting the distribution of individual percentage scores for the Pairs Matching Task across the control group, DPs, and SRs

Using the total number of correct responses (i.e. correct hits for matched pairs and correct rejections for mismatched pairs), the overall percentage accuracy was calculated for each participant. Figure 5 illustrates the distribution of percentage accuracy across the different participant groups. Overall, performance ranged from 35.4 to 100% accuracy (*M* = 67.65%, SD = 14.32). A between-subjects ANCOVA, with participant age entered as a covariate, comparing participant groups (control, DP, SRs) was performed. Once again, the covariate of participant age was significant, *F*(2,109) = 8.07, *p* = 0.005, *ƞp*^2^ = 0.069, BF_10_ = 1.93. The analysis also revealed an effect of group, *F*(2,109) = 88.30, *p* < 0.001, ƞp^2^ = 0.62, BF_10_ = 6.93 × 10^19^. Follow-up tests indicated that SRs achieved a higher performance accuracy (M = 84%, SD = 7.15) compared to DPs (*M* = 57%, SD = 9.45), *t*(62) = 12.99, *p* < 0.001, *d* = 3.25, and controls (*M* = 62%, SD = 9.68), *t*(80) = 10.89, *p* < 0.001, *d* = 2.45. In turn, controls also performed better than DPs on this task, *t*(78) = 2.55, *p* = 0.013, *d* = 0.59.

#### Response times

Complementary analysis of mean reaction times were conducted next. A between-subjects ANCOVA, with participant age entered as a covariate, comparing participant groups (control, DP, SRs) was performed. This analysis did not find an effect of participant age, *F*(1,109) = 1.18, *p* = 0.280, *ƞp*^2^ = 0.01, BF_10_ = 1.93. An effect of participant group was found, *F*(2,109) = 26.17, *p* < 0.001, *ƞp*^2^ = 0.32, BF_10_ = 6.932 × 10^19^. Post hoc independent samples *t*-tests revealed that SRs responded slower (*M* = 5843 ms, SD = 2361) compared to DPs (*M* = 4057 ms, SD = 1864), *t*(62) = 3.35, *p* = 0.001, *d* = 0.84, and controls (*M* = 2588 ms, SD = 1742),* t*(80) = 7.18, *p* = 0.001, *d* = 1.62. DPs were also slower compared to controls, *t*(78) = 3.58, *p* < 0.001, *d* = 0.82.

### Relationship between ACT, PMT and participant age

Next, we examined the relationship between accuracy on the Age Classification Task, Pairs Matching Task, and participant age. The analysis was performed for all groups combined, and separately for each group to avoid the outcome being skewed by the more extreme scores. A Shapiro–Wilk test revealed a violation of normality for the control group, *W* = 0.85, *p* < 0.001, and all groups combined, *W* = 0.95, *p* < 0.001, therefore non-parametric Spearman’s correlation was performed for these analysis, and Pearson’s correlation for DPs and SRs. For the control groups, this analysis revealed a moderate positive correlation between percentage accuracy for ACT and PMT scores, *r*(48) = 0.49, *p* < 0.001, but not for DPs, *r*(31) = 0.05, *p* = 0.796, or SRs, *r*(33) = 0.27, *p* = 0.116. A significant positive correlation was found when all groups were included in the correlation, *r*(113) = 0.35, *p* < 0.001.

For all the groups combined, we also performed a Spearman’s correlation between participant age and ACT which revealed a weak negative relationship, *r*(48) = − 0.23, *p* = 0.012. These data suggest that as participant age increases performance on the age classification task decreases. To exclude the possibility that the relationship between the ACT and PMT is being unduly influenced by participant age, we reran the Spearman’s correlation but this time we statistically controlled for participant age. This did not change the relationship between performance on the ACT and PMT in the combined groups, *r*(113) = 0.31, *p* < 0.001.

## Discussion

Study 3 compared performance accuracy of controls, DPs, and SRs on an age classification task. Findings demonstrate that SRs were more accurate in distinguishing between faces which were over and under the age of 18 compared to both the DPs and controls. The accuracy of the DPs was on par with controls, however controls responded on average 500 ms faster than both DPs and SRs. This indicates that there may have been a speed-accuracy trade-off, and these quicker responses could have led to more errors. Slower RTs for both SRs and DPs compared to controls is an unexpected finding. However, given that participants were not asked to respond as quickly as possible, but rather to focus on accuracy, we do not expect response time to have disproportionately affected accuracy, but it may reveal some nuances in processing time. A similar pattern was also mirrored in the face matching task (PMT), such that the control group were the fastest responders, followed by DPs, and finally SRs.

Accuracy is often the measure of focus in face perception research, response times and potential speed-accuracy trade-offs are not routinely reported (Fysh & Ramon, 2022). Thus, our ability to draw direct comparisons with other face processing tasks between SRs, DPs, and controls is limited. Nonetheless, some differences in RTs can be found in face matching research and have been related to factors such as processing time, for example, DPs are typically slower than controls on a range of face processing tasks (e.g. Delvenne et al., 2004; Jansari et al., 2015; Murray et al., 2022). Alternatively, RT differences may arise from motivational differences, for example, passport officers recorded lower RTs than the control group on a face matching task (White et al., 2014).

The results also indicate that there is a positive correlation between performance on the Age Classification Task and the Pairs Matching test for the control group and all groups combined, which suggests that processes underlying these two tasks may be linked. No correlation was found when the correlational analysis was performed separately for DPs or SRs. We also excluded the possibility that this relationship can be accounted for by participant age.

## General discussion

Few studies have examined the potential link between age and identity perception, despite the theoretical and applied implications. Three separate age tasks were administered to begin to probe for age perception differences across groups with different facial recognition abilities (i.e. DPs, SRs, and age-matched controls), and to tap into potential differences in age perception processes across different tasks. The studies comprised: (1) numeric estimation of faces across the life span, (2) numeric estimation of faces across the life span with added noise-distortion, and (3) age classification of faces as under- and over- the age of 18 and a face matching task. An interaction between age and identity perception abilities emerged for the age classification task but this relationship did not extend to the numeric estimation tasks. These distinct differences in performance patterns suggest that a complex and nuanced relationship may exist between the perception of age and facial identity.

For studies 1 and 2, two measures of age estimation were calculated. The first was *Estimation Bias* and, rather than a measure of accuracy, this measure detects *systematic* patterns of over- or under-estimation of age. Thus, an average score which is close to zero is not a reflection of accuracy, but rather reflects an absence of a systematic pattern of either over- or under- estimating age. Across both studies we find that the age of younger faces tends to be more overestimated, and this gradually reduces as the age category of the face increases, with faces in their 60 s recording negligible bias. The pattern of younger faces being over-estimated is in line with previous research (e.g. Clifford et al., 2018; Davis & Attard-Johnson, 2022; Thorley, 2020; Willner & Rowe, 2001). Although the reason for this is not fully understood, various explanations for this trend have been put forward. One prominent explanation relates to the presence of serial dependence effects whereby the age estimate of the current face is influenced by the face estimated on the previous trial (Clifford et al., 2018). However, given the full randomisation of presentation of the trials across all participants, and that a similar or opposite pattern was not observed for older faces, it is unlikely that serial dependence explains the systematic upward bias for younger faces recorded in these studies.

This upward bias of younger faces is even more pronounced when the image quality is reduced via noise-distortion (Study 2), such that the over-estimation for faces in their 20 s and 30 s is much higher to start with (around 7 years as opposed to 3 years). Nonetheless, this bias reduces with increasing age, except for SRs, and to a lesser extent controls, who continue to overestimate faces in their 50 s by around 6 years (compared to 4 and 0 years in controls and DPs, respectively). It is possible that this difference is exaggerated when the noise-distortion is added because the distortion disrupts or masks the visibility of smooth skin texture, luminance, contrast, and skin tone. Information relating to facial skin tone and texture is considered critical (Porcheron et al., 2013) and contributes between 25 and 33% of the information in the assessment of age (González-Alvarez & Sos-Pena, 2023).

However, Estimation Bias does not capture the full picture. This is because inaccuracy in estimation may be concealed if participants in the group recorded inconsistent under-and over-estimates. Such inconsistency would lead to the estimates cancelling out each other. For this reason, a second measure is calculated, *Absolute Accuracy.* This measure omits the direction of deviation and simply takes the average difference between estimation and actual age. When we use this measure with the high- quality images (Study 1), we find that the average deviation from the true age across all stimulus ages was 6.5 years with individual means ranging from 1 to 16 years. However, there were no differences across any of the three participant groups or age categories. For the noise-distorted images (Study 2), the average deviation was slightly higher, 7.7 years, with individual deviations in estimates from true age ranging from 1 to 22.5 years. There were some small differences between the stimulus age categories, but there were no differences between the SRs, DPs, and controls. The wide range in responses indicates that there are individual differences in numeric estimation ability, but these could not be accounted for with face processing ability.

These initial findings suggest that in terms of numeric estimation, DPs, SRs and controls perform similarly overall. Given that some evidence suggests that DPs have difficulty accessing internal facial representations due to a disconnection between the memory stores and perceptual and semantic systems (Bate et al., 2008; Fox et al., 2008), we expected that DPs may be unable to use stored representations for other aspects of face processing, including age. However, this notion was not supported here. One possible explanation is that storing and accessing age-related information is independent to storing and accessing facial identity representations. On the same lines, it is also plausible that mental representation of age does share storage with facial memory but forms an independent route or connection with perceptual or semantic systems which remains unaffected. An alternative explanation is that numerical estimation of age utilises processes related to cognitive estimation abilities (e.g. estimating distance, size, and duration) more than face perception and recognition abilities. Cognitive estimation draws on a set of higher-order cognitive functions within Executive Functioning (EF) which is used to guess an answer sometimes guided by knowledge or previous experience from semantic memory (Shallice & Evans, 1978; Wagner et al., 2011).

Therefore, it is possible EF abilities and semantic memory are the primary processes involved in completing this task and the reliance on face perception abilities is minimal or offset. If this is the case, then it could explain why DPs perform just as well as controls and SRs since EF is not considered to be impaired in this population. In fact, given that EF is used to extract knowledge from situational context to problem solve (Suchy, 2009), it may even be possible that EF is enhanced in DPs who regularly use context cues as a coping strategy for managing the day-to-day difficulties with face recognition (e.g. Adams et al., 2020; Murray et al., 2018; Portch et al., 2023). Indeed, greater activation in the prefrontal cortex of a small number of DPs has been previously recorded in an fMRI study (Avidan et al., 2005). Although the prefrontal cortex is associated with Executive Functions (Yuan & Raz, 2014), the direct link between EF and face processing in DPs has not yet been examined.

The *numeric estimation* aspect of the task may therefore be compensating for face and age processing deficits. Therefore, to eliminate the cognitive estimation aspect of the task, and to test performance using a more ecologically valid paradigm closer to a set-up in a real-world professional setting, we conducted a final study with a greater focus on perceptual skills. In Study 3 participants decided whether a face between the age of 14 and 22 was over- or under- the age of 18. On this task, performance of SRs was 4% more accurate than that of controls and DPs, and a positive correlation was found between ACT and the face perception task (Pairs Matching Test). These data suggest, for the first time, that SRs may have a unique ability which allows them to perform better on this task than even controls. Conversely, DPs were not negatively affected by their face recognition deficit when compared to controls. The lack of impairment found in DPs is consistent with a small set of studies, using different methodological approaches, demonstrating that the ability to accurately perceive age is unaffected in DPs (Chatterjee & Nakayama, 2012; Dobel, 2007). There is caveat here. A closer look at reaction time data suggests that a speed/accuracy trade-off may have occurred for the control group. Specifically, the control group responded 500 ms faster than both the DPs and the SRs which may have impeded their performance to the level of DPs. Further research will be needed to examine these differences in more depth.

Taken together, these findings are the first to demonstrate a link between processing of facial age and identity which differs depending on the specific task being performed. Although numeric estimation and classification of age both require the observer to perceive age, differences between participant groups only emerged for the task requiring a binary classification of age, and only for SRs. This suggests that the relationship may be partial or limited to only a specific aspect of face processing and only for people who have exceptional face identification abilities (SRs). It is possible that the differences are occurring at the early stages of face processing. Differences in visual processing strategies of SRs and DPs have been found to explain some of the differences in face recognition ability (Tardif et al., 2019) and therefore may also affect acquisition of age information from facial features. SRs and DPs use the same critical features for face recognition as the typical population (Abudarham et al., 2021), but there are differences in time spent viewing specific regions (Bobak et al., 2017) and DPs generally demonstrate impaired processing of the eyes (Bobak et al., 2017; Tardif et al., 2019). Further research is needed to directly examine whether differences in use of facial information affects age processing. Alternatively, differences may be occurring during later stages of processing.

### Limitations

There are some caveats to consider. Age perception research has found an age-bias which could have affected findings in this study (Moyse et al., 2014; Willner & Rowe, 2001). However, precautions were taken to reduce the influence of participant age, specifically the participant groups were matched in age and participant age was included as a covariate in the analysis. This analysis demonstrated that participant age was an influencing factor but was statistically excluded from the analysis. It is also possible that performance on the age classification task which comprised 14–22-year-olds, and the numeric estimation task which comprised 20–70-year-olds, recorded variances in performance because of the differences in age ranges. However, images in the numeric estimation task were also broken down by stimulus age, and no differences between groups were found for the younger age group (i.e. the 20- and 30-year-olds). Nonetheless, further work could consider transforming the stimuli used from the age classification task to a numeric estimation task and making a direct comparison.

The reliability analysis for the two numeric estimation tasks was found to be good. However, there was only moderate internal consistency found for the age classification task. While this is a reasonable starting point, more work is needed to improve on this and develop it further for use as an assessment tool. Further refinement could be achieved by increasing the number of trials, and closely examining each image to determine whether some faces were rarely correctly classified in the ACT. However, producing a validated assessment tool was beyond the scope of this manuscript, but rather the aim was to provide a starting point.

## Conclusion

Differences in performance on age perception between DPs, SRs, and controls emerged for the age-classification task but not for the numeric estimation of age tasks, and the only difference was with regard to the SRs who performed better than DPs and controls. Thus, the relationship between age processing and identity perception is unclear and may be complex, but this work offers a starting point for further investigation. These findings raise several questions around the specific cognitive processes are involved in different age estimation tasks, and whether SRs and DPs differ on some of the cognitive processes involved and not others.

## Significance statement

Our study examines the interplay between facial age perception and facial identity recognition by comparing ‘super-recognisers’, individuals with developmental prosopagnosia (SPs), and neurotypical controls on a series of age perception paradigms. Our findings demonstrate that differences in age perception are not uniform across all paradigms, suggesting nuanced differences in the way age estimation ability is captured. Findings also hint at a possible relationship between age processing and facial identity recognition, though this warrants further investigation. Understanding the nuances of age perception can help to improve processes for estimating age in applied professional and security settings (e.g. sales of restricted items, classification of asylum as minors or adults, and assess online explicit materials for minors), as well as broader reaching implications in other areas, such as for improving facial recognition technology.

## Supplementary Information

Supplementary Material 1.

## Footnotes

1 Mauchley’s test of sphericity showed that the assumption of sphericity was violated. The analysis was also performed using Green-House Geisser correction. There were no changes to the patterns, therefore the uncorrected analyses are reported.
