# Supplementary material for: Different facets of age perception in people with developmental prosopagnosia and “super-recognisers”
Source: Cogn Res Princ Implic. 2024 Nov 13;9:76. doi: 10.1186/s41235-024-00603-4 (PMC11557776; doi:10.1186/s41235-024-00603-4)
Supplement: Supplementary file 1 — Supplementary Material 1. [file 41235_2024_603_MOESM1_ESM.docx]

**Supplementary Materials**

**Table 1: Performance on face recognition and perception tasks for the SR participants.** For all tasks, 1.96 SD cutoffs are applied as per Bate et al. (2018), together with the requirement for significantly superior performance on at least two of the three tasks. Note that three participants did not complete the MMT, but exceeded threshold on the CFMT+ and PMT. All control norms are taken from Bate et al. (2018).

|  | Age | Sex | CFMT+^1^  (total=102; cutoff = 90) | MMT^2^  (total=90; cutoff=73) | PMT^3^  (total=48; cutoff=40) |
| --- | --- | --- | --- | --- | --- |
| SR02 | 39 | male | 98 | 89 | 41 |
| SR03 | 52 | female | 92 | 64 | 40 |
| SR07 | 34 | female | 92 | 81 | 40 |
| SR08 | 29 | male | 96 | 86 | 40 |
| SR100 | 23 | female | 98 | 81 | 45 |
| SR102 | 31 | female | 93 | 69 | 41 |
| SR106 | 37 | male | 93 | 73 | 46 |
| SR110 | 41 | female | 95 | 86 | 39 |
| SR113 | 35 | female | 95 | 69 | 40 |
| sr114 | 35 | male | 95 | 88 | 40 |
| SR115 | 35 | female | 96 | 85 | 43 |
| SR116 | 43 | female | 91 | No score | 42 |
| SR119 | 31 | male | 97 | 87 | 45 |
| SR120 | 36 | female | 93 | 78 | 47 |
| SR15 | 50 | female | 92 | 74 | 41 |
| SR16 | 38 | male | 93 | 72 | 40 |
| SR18 | 55 | female | 98 | 64 | 40 |
| SR19 | 41 | male | 96 | 74 | 38 |
| SR21 | 52 | female | 94 | 82 | 45 |
| SR26 | 63 | female | 96 | 85 | 41 |
| SR36 | 28 | female | 92 | No score | 40 |
| SR47 | 39 | female | 90 | 75 | 36 |
| SR51 | 48 | female | 98 | 78 | 41 |
| SR52 | 53 | female | 100 | 76 | 44 |
| SR54 | 60 | male | 99 | 74 | 34 |
| SR56 | 43 | male | 95 | 75 | 41 |
| SR63 | 41 | female | 90 | 79 | 44 |
| SR65 | 30 | female | 101 | 78 | 41 |
| SR77 | 52 | female | 99 | 74 | 37 |
| SR78 | 48 | female | 96 | 84 | 40 |
| SR89 | 56 | female | 94 | No score | 46 |
| SR93 | 41 | female | 95 | 74 | 37 |
| SR95 | 35 | male | 96 | 81 | 38 |

^1^Cambridge Face Memory Test – Extended Form (Russell et al., 2009).

^2^Models Memory Test (Bate et al., 2018).

^3^Pairs Matching Test (Bate et al., 2018).

**Table 2: Performalece on face recognition and perception tasks for the DP participants.**

|  | Age | Sex | CFMT  (total=72; cutoff=46^1^) | CFPT  (no. errors; cutoff=58^2^) | Famous Faces  (% correct; cutoff=80.00%^3^) |
| --- | --- | --- | --- | --- | --- |
| DP09 | 56 | female | 35 | 66 | 18.75 |
| DP100 | 43 | female | 45 | 24 | 65.38 |
| DP104 | 45 | female | 44 | 40 | 61.54 |
| DP109 | 51 | female | 41 | 48 | 76.92 |
| DP110 | 47 | female | 38 | 40 | 65.38 |
| DP115 | 57 | male | 25 | 72 | 44.00 |
| DP122 | 33 | female | 35 | 80 | 81.82 |
| DP19 | 52 | female | 42 | 66 | 65.91 |
| DP23 | 51 | male | 38 | 72 | 61.67 |
| dp25 | 53 | female | 39 | 62 | 67.30 |
| DP27 | 52 | female | 32 | 66 | 46.43 |
| DP29 | 61 | female | 35 | 72 | 81.40 |
| DP30 | 39 | female | 33 | 48 | 38.89 |
| dp32 | 25 | female | 33 | 66 | 39.22 |
| DP34 | 55 | female | 35 | 70 | 25.00 |
| DP35 | 29 | female | 31 | 48 | 31.48 |
| DP41 | 61 | female | 44 | 46 | 40.43 |
| DP42 | 59 | female | 39 | 60 | 63.79 |
| dp43 | 58 | male | 36 | 58 | 31.67 |
| DP44 | 25 | female | 38 | 86 | 57.41 |
| DP47 | 55 | female | 45 | 60 | 43.86 |
| DP48 | 34 | female | 44 | 50 | 67.27 |
| DP51 | 24 | female | 38 | 48 | 49.15 |
| DP60 | 59 | female | 46 | 38 | 15.52 |
| DP70 | 50 | female | 41 | 46 | 46.00 |
| DP79 | 55 | male | 43 | 76 | 52.00 |
| DP80 | 51 | male | 46 | 60 | 55.00 |
| DP86 | 22 | female | 32 | 50 | 60.00 |
| DP87 | 38 | female | 36 | 32 | 53.70 |
| DP92 | 38 | female | 41 | 74 | 49.06 |
| DP98 | 48 | female | 31 | 48 | 50.00 |

*Note*. To be included in the DP sample, participants were required to self-report severe difficulties in everyday face recognition and to show impaired performance (at least 1.7 SDs from the control mean, e.g., DeGutis et al., 2014, Murray et al., 2022) on at least two of three objective screening tasks (for recent discussion about the benefits of using more liberal inclusion criteria in the diagnosis of DP see DeGutis et al., 2023; Burns et al., 2022). Participants also scored within the typical range on the Autism Quotient (Baron-Cohen et al., 2001). All individuals included here also meet alternative preferences for diagnosis, where only scores on face memory tasks (i.e., the CFMT and Famous Faces Test) are considered and not those on perceptual tasks (i.e., the CFPT).

^1^ The Cambridge Face Memory Test (Duchaine & Nakayama, 2006); 1.7SD cut-off score of 46/72 (norms from the age-matched controls of Duchaine, Yovel & Nakayama, 2007).

^2^ The Cambridge Face Perception Test (Duchaine, Germine & Nakayama, 2007); 1.7SD cut-off score of 58 (norms from the age-matched controls of Duchaine, Germine & Nakayama, 2007). Note that scores represent the number of errors, such that higher values indicate poorer performance.

^3^ Famous Face Test (Bate et al., 2019); 1.7SD cut-off score of 80% (norms from the age-matched controls of Bate et al., 2019).

**References**

Baron-Cohen, S., Wheelwright, S., Skinner, R., Martin, J., & Clubley, E. (2001). The Autism-Spectrum Quotient (AQ): Evidence from Asperger Syndrome/High-Functioning Autism, males and females, scientists and mathematicians. *Journal of Autism and Developmental Disorders, 31*, 5–17.

Bate, S., Bennetts, R., Gregory, N.J., Tree, J., Murray, E., Adams, A., Bobak, A.K., Penton, T., Yang, T., & Banissy, M. (2019). Objective patterns of face recognition deficits in 165 adults with self-reported developmental prosopagnosia. *Brain Sciences, 9*, 133.

Bate, S., Frowd, C., Bennetts, R., Hasshim, N., Murray, E., Bobak, A. K., Wills, H., & Richards, S. (2018). Applied screening tests for the detection of superior face recognition. *Cognitive Research: Principals and Implications, 3*, 22.

Burns, E.J., Gaunt, E., Kidane, B., Hunter, L., & Pulford, J. (2022). A new approach to diagnosing and researching developmental prosopagnosia: Excluded cases are impaired too. *Behavior Research Methods*. https://doi.org/10.3758/s13428-022-02017-w.

DeGutis, J., Bahierathan, K., Barahona, K., Lee, E., Evans, T.C., Shin, H.M., Mishra, M., Likitlersuang, J., & Wilmer, J.B. (2023). What is the prevalence of developmental prosopagnosia? An empirical assessment of different diagnostic cutoffs. *Cortex, 161*, 51-64.

DeGutis, J., Cohan, S., & Nakayama, K. (2014). Holistic face training enhances face processing in developmental prosopagnosia. *Brain, 137*, 1781-98.

Duchaine, B.C., Germine, L., & Nakayama, K. (2007). Family resemblance: Ten family members with prosopagnosia and within-class object agnosia. *Cognitive Neuropsychology, 24*, 419– 430.

Duchaine, B.C., & Nakayama, K. (2006). The Cambridge face memory test: Results for neurologically intact individuals and an investigation of its validity using inverted face stimuli and prosopagnosic participants. *Neuropsychologia, 44*, 576– 585.

Duchaine, B.C., Yovel, G., & Nakayama, K. (2007). No global processing deficit in the Navon task in 14 developmental prosopagnosics. *Social Cognitive and Affective Neuroscience, 2*, 104– 113.

Murray, E., Bennetts, R., Tree, J., & Bate, S. (2022). An update of the Benton Facial Recognition Test. *Behavior Research Methods, 54*, 2318-2333.

Russell, R., Duchaine, B., & Nakayama, K. (2009). Superrecognizers: People with extraordinary face recognition ability. *Psychonomic Bulletin and Review, 16*, 252–257.
